# Spanish version of SPADI (shoulder pain and disability index) in musculoskeletal shoulder pain: a new 10-items version after confirmatory factor analysis

**DOI:** 10.1186/s12955-016-0436-4

**Published:** 2016-03-01

**Authors:** Alejandro Luque-Suarez, Antonio Rondon-Ramos, Manuel Fernandez-Sanchez, Kathryn E. Roach, Jose Miguel Morales-Asencio

**Affiliations:** Department of Physical Therapy, University of Malaga, Malaga, Spain; Las Lagunas Primary Health Care Center, Costa del Sol Sanitary District, Fuengirola-Mijas, Spain; Department of Nursing, Physical Therapy and Medicine, University of Almeria, Almeria, Spain; Department of Physical Therapy, Miller School of Medicine, University of Miami, Miami, USA; Department of Nursing, University of Malaga, Malaga, Spain

**Keywords:** Shoulder pain, Diagnostic techniques and procedures

## Abstract

**Background:**

The Shoulder Pain and Disability Index (SPADI) is a tool designed to evaluate the impact of shoulder pathology. The aim of this study was to cross culturally adapt a Spanish version of the SPADI for Spanish population with a musculoskeletal shoulder pain, and to determine the psychometric properties of this instrument using confirmatory factor analysis (CFA).

**Methods:**

Cross-cultural adaptation was performed according to the international guidelines. To assess factor structure, a confirmatory factor analysis was done. Internal consistency was measured using Cronbach’s alpha. Item-total and inter-item correlations were assessed. Pearson and Spearman correlations were calculated to assess the convergent validity between SPADI and quick-DASH.

**Results:**

A new Spanish version of SPADI was achieved. The original SPADI factor structure was tested by CFA, obtaining a poor fit: relative chi-square (*χ*2/df) 3.16, CFI 0.89, NFI 0.92, and RMSEA 0.10 (90 % CI 0.08 to 0.12). An additional model was tested, after deleting items which have had a poor adjustment in the model (1, 11, and 12), obtaining the best fit: relative chi-square (*χ*2/df) of 1.94, CFI 0.98, NFI 0.95, GFI 0,95, and RMSEA 0.06 (90 % CI 0.04 to 0.09). The analysis confirmed the bidimensional structure (pain and disability subscales). A correlation Spearman’s Rho coefficient of 0.752 (*p* < 0.0001) and a Cronbach’s alpha of 0.90 were obtained.

**Conclusions:**

This study validated a new 10-items version of SPADI for Spanish population with musculoskeletal shoulder pain providing a patient reported outcome measure that could be used in both clinical practice and research.

## Background

Shoulder pain is one of the most common musculoskeletal conditions seen in primary care [1], after low back and neck pain. It affects one in three adults [2, 3], accounting 1 % of General Practice consultations in primary care [4]. In working-age populations, the prevalence of shoulder pain associated with musculoskeletal disorders is even higher [5], and increases with age [6].

Shoulder disorders are frequently accompanied by pain and restricted shoulder movement leading to difficulties in performing certain activities. Recent research suggests that shoulder pain not only affects function during work and leisure time activities, but also may interfere with psychological and social wellbeing [7]. Additionally, environmental factors, such as psychological distress, may contribute to the development of chronic shoulder problems [8].

A variety of musculoskeletal pathologies can cause shoulder pain including subacromial syndrome, frozen shoulder, rotator cuff tendonitis and tear, calcyfying tendonitis, biceps large portion tendonitis, and tear and gleno-humeral instability [9].

The impact of shoulder disorders can be assessed in different ways. Traditionally, the assessment has focused on the impairments associated with shoulder pathology by evaluating the range of motion, strength, or pain [10]. However, patients are more concerned with the activity limitations that result from these impairments. This has lead to an increasing emphasis on patient reported outcome (PRO) measures.

The Shoulder Pain and Disability Index (SPADI) is a PRO measure that was developed for use in an outpatient setting. It was designed to measure the impact of shoulder pathology in terms of pain and disability, for both current status, and change on status over time [11]. The SPADI is a self-administered questionnaire that consists of two dimensions, one for pain and the other for functional activities [11]. The pain dimension consists of five questions regarding the severity of an individual’s pain. Functional activities are assessed with eight questions. The SPADI takes 5 to 10 min for a patient to be completed. To answer the questions, subjects place a mark on a “0 to 10” numbered scale for each question. Verbal anchors for the pain dimension are ‘no pain at all’ and ‘worst pain imaginable’, and those for the functional activities are ‘no difficulty’ and ‘so difficult it required help’. The scores from both dimensions are averaged to produce a total score [11].

The SPADI has been used for measuring the outcomes in different studies and shoulder conditions, such as shoulder pain, various upper extremity diagnoses, various shoulder diagnoses, adhesive capsulitis, rotator cuff, after shoulder arthroplasty, total shoulder arthroplasty, various shoulder surgery, and in different populations and clinical settings, as orthopedic practice, outpatient physiotherapy and community volunteers [12].

The SPADI has been shown to be valid as a measure of pain and disability in community-based patients reporting shoulder pain due to musculoskeletal pathology. The SPADI has good internal consistency with a Cronbach’s alpha of 0.95 for the total score, 0.92 for the pain subscale and 0.93 for the disability subscale. The SPADI has also shown ability to detect change over time [13]. In comparing a number of shoulder specific questionnaires in primary care, the SPADI and SRQ (shoulder questionnaire rating) were found to be the most sensitive to detecting change and the SPADI required the least time to complete [14]. Another study reviewing the clinimetric properties of several shoulder questionnaires concluded that SPADI had good construct validity (>0.74) [15].

The increased number of international research projects as well as the diversity of populations and cultures living in a same region, has created the need to validate PRO measures in groups different from those originally used to develop the measure. This requires both translation into a new language and accommodation for differences in cultural characteristics [16].

To our knowledge, only one study has attempted to validate a Spanish language version of the SPADI and this study was conducted in women with shoulder pain following breast cancer surgery rather than shoulder pain due to musculoskeletal pathology [17]. Furthermore, no confirmatory factor analysis has been carried out for any of the multiple versions of SPADI. There is a need of constant update for any PRO measure to guarantee that the mentioned PRO retains all its psychometric properties and its equivalence between original and translated versions, as well as to evaluate its performance in other contexts [18]. This process should be carried out with robust methods, such as confirmatory factor analysis, to test the hypothesis of its original dimensionality. Moreover, the validity of a PRO need to be tested to assess whether its validity is dependent on the population in which the instrument was originally validated, as clinimetric properties many times depend on situational circumstances [18]. Therefore, additional testing in specific populations, i.e., musculoskeletal shoulder pain disorders, need to be carried out.

Hence, the aim of this study is twofold: 1) to translate and validate a Spanish version of the SPADI for Spanish population with a broad range of shoulder disorders and, 2) to determine the psychometric properties of this instrument, using confirmatory methods.

## Methods

### Patients and design

This study was approved by the Ethics Committee of Costa del Sol, March 2014, Spain. All participants in the study gave a written informed consent. Participants were recruited from six primary health care centres in the province of Malaga, Spain. Participants met the following inclusion criteria: i) shoulder pain, defined as “pain in the shoulder region brought on or exacerbated by movement at that shoulder”. ii) aged between 18 and 80 years, iii) first language was Spanish (Spain), iv) able to read written Spanish. Participants were excluded from the study if they did not have the capacity to comprehend the questionnaire due to cognitive or emotional impairment. Prior to conducting study, the authors obtained permission for the original author (Dr KE Roach), who was also involved in the study.

### The study consisted of two phases

The study was conducted in two phases. The first was thehe cross-cultural adaptation of the SPADI and the second was the validation of the adapted SPADI. The cross-cultural adaptation process was undertaken using the guidelines and methodology recommended by the International Society for Pharmacoeconomics and Outcomes Research (ISPOR) for the translation and validation of patients reported outcome measures [19]. The validation of the adapted SPADI was then undertaken by examining its psychometric properties and conducting a confirmatory factor analysis.

### Cross-cultural adaptation

Cross-cultural adaptation involved eight stages: (1) *forward translation*, (2) *reconciliation*; (3) *back translation*, (4) *back translation review*, (5) *harmonisation*, (6) *pilot testing/cognitive debriefing*, (7) *pilot testing review/review of cognitive debriefing results* and (8) *proofreading*.

#### Forward translation

Two forward translations in Spanish were undertaken from the original English language version of the SPADI. The translations were undertaken by two independent health professionals who were native residents of Spain and fluent in both Spanish and English.

#### Forward translation reconciliation

The two forward translations were reconciled into one version (draft 1) by the two original translators, a third independent translator, and with additional input from the project lead.

#### Back translation

Two professional English native translators residing in Spain back translated the reconciled Spanish language version (draft 1) into English independently. The translators had neither prior knowledge of the SPADI nor of the original wording of the English version of the SPADI.

#### Back translation review

The principal investigator and a native Spanish speaker fluent in both languages reviewed the back translation for any discrepancies in meaning or terminology used. Any problematic item was discussed until the discrepancies were resolved. This process resulted in a refined second draft of the Spanish translation (draft 2).

#### Harmonisation

To produce the final Spanish language translation, a harmonisation meeting was undertaken involving three Spanish translators, the senior investigator and the developer of the original USA version of the SPADI. During this meeting, any discrepancies or issues that were highlighted from the back translation were discussed, the translated version of the SPADI was evaluated and a final version agreed.

#### Pilot testing/cognitive debriefing

Once the translation process was completed, the translation was formatted to match precisely the original American language version. The translated SPADI version was initially assessed for comprehensibility in five patient participants, who were Spanish residents and native speakers, met the inclusion criteria described above and had a low educational background without being illiterate. At this stage, each participant was asked by the in-country investigator to carry out the following tasks:

- To complete a copy of the translated SPADI and time needed.
- To comment on the response options within the back-translated SPADI.
- To comment on any wording that was difficult to understand.
- To suggest alternative wording/phrasing for any wording that was difficult to understand.
- To describe in their own words what the wording meant to them. These responses were recorded verbatim and translated into English. The five patients’ responses were summarised by the senior investigator. This summary also contained any changes, recommendations or suggestions indicated by the participants and in-country investigators.

#### Pilot testing review/review of cognitive debriefing results

To improve the performance of the translated questionnaire, the pilot testing results were reviewed by the in-country investigators. At this stage, any item that caused comprehensibility difficulties for more of the 40 % of the participants was reviewed, and any modifications suggested by the respondent’s comments were incorporate to the final translated version.

#### Proof-reading

The senior investigator and a translator, who was not involved in the translation process previously, independently proofread the final formatted translation, and any suggested changes were discussed with the senior investigator. Furthermore, the Flesh Reading Ease test and the Flesh Kincaid Grade Level were calculated for readability [20].

Following this process, a final draft of the SPADI translated and culturally adapted into ‘Spanish for Spain’ was locked down and entered into the cross-cultural validation phase (final draft).

### Validation phase

Before completing the questionnaire, the following data were recorded: age, sex, professional status, education level, affected shoulder and diagnosis. The questionnaires were administered by physical therapists working in the six different physical therapy rooms. They addressed any possible concern of the subjects. Items were numbered from 1 to 13. Items 1 to 5 were from pain subscale and items 6 to 13 were from disability subscale.

#### Sample size calculation

To test a two-factor model, assuming the null hypothesis of a mean square error of approximation (RMSEA) from 0.04 to 0.085, with an alpha value of 0.05, a statistical power of 0.8 and a maximum of 26 degrees of freedom, as suggested by MacCallum et al [21], sample of 196 subjects was required which was over-estimated by 10 % to cover possible losses. The calculations were performed with the Statistica 12 software [22].

#### Data analysis

Descriptive statistics were carried out with means, standard deviations, and absolute and relative frequencies. Analysis of normality of distributions was evaluated by the Kolmogorov-Smirnov test, symmetry analysis and kurtosis. Internal consistency was calculated using Cronbach’s alpha. A Cronbach’s alpha between 0.70 and 0.95 was considered “good” [23]. Moreover, item-total and inter-item correlations were assessed. Pearson and Spearman correlations were calculated to assess the convergent validity between SPADI and quick-DASH-Spanish version [24]. To assess factor structure, a confirmatory factor analysis was done, the evaluated model was fit with the following parameters: the penalizing function (chi square/ df), which is indicative of good fit with values less than 3; Root Mean Square Error of Approximation (RMSEA) and confidence intervals (CI 90 %), taking the value 0.05 as cut-off of good fit; Normed Fit Index (NFI), the Comparative Fit Index (CFI), and Goodness of Fit Index (GFI) with a minimum value of good fit of 0.90. Multinormality was evaluated with the Mardia’s coefficient (multivariate curtosis), which could not be over “p” (p + 2), where “p” are the number of observed variables [25]. All the analyses were performed with SPSS 21 [26] and AMOS 21 [27].

## Results

### Traslation and cross-cultural adaptation process

Once a definitive back translation was obtained, the original author (Dr Kathryn Roach) reported some inconsistencies between the translated and original versions. To solve this issue, the expert committee was met, and new items were developed in the final Spanish version.

In the pilot testing phase, results showed no discrepancies in meaning or terminology used in the translated version of the SPADI. Hence, no modification of this version was done. Subjects did not request assistance in interpretating of the questionnaire or any of its items. The time needed to fill out the questionnaire was 4.61 min (SD 0.99). The result for Flesh Reading Ease test was 56.7, and 7.6 for the Flesh Kincaid Grade Level.

### Validation phase

The final sample consisted of 219 participants, of which, 34.7 % were male and 65.2 % female, with a mean age of 55.08 (SD: 13.63). Characteristics of the sample and their clinical status are described in Table 1.

**Table 1:** Characteristics of participants

		Female (*n* = 143)	Male (*n* = 76)	*p*
Age (years)		56.27 (SD 13.27)	52.75 (SD 14.10)	0.073
Professional status n (%)	Active	42 (52.5)	38 (47,5)	0.001
	Unemployed	23 (74,19)	8 (25,81)	
	Sick-leave	19 (67,86)	9 (32,14)	
	Retired	37 (63,79)	21 (36,21)	
	House-wife	22 (100)	0 (0)	
Educational level n (%)	Low	56 (73,68)	20 (26,32)	0.247
	Medium	50 (60,98)	32 (39,02)	
	High	30 (58,82)	21 (41,18)	
	Illiteracy	4 (57,14)	3 (42,86)	
Affected shoulder n (%)	Dominant	75 (60.50)	49 (39.50)	0.118
	Non-dominant	69 (71.10)	28 (28.9)	
Diagnosis n (%)	Shoulder pain	36 (63,16)	21 (36,84)	0.505
	Frozen shoulder	11 (84,62)	2 (15,38)	
	Subacromial	19 (70,37)	8 (29,63)	
	Tendon	29 (63,04)	17 (36,96)	
	Surgery	26 (59,09)	18 (40,91)	
	Fracture	11 (68,75)	5 (31,25)	
	Instability	0 (0)	1 (100)	

**Fig. 1:**
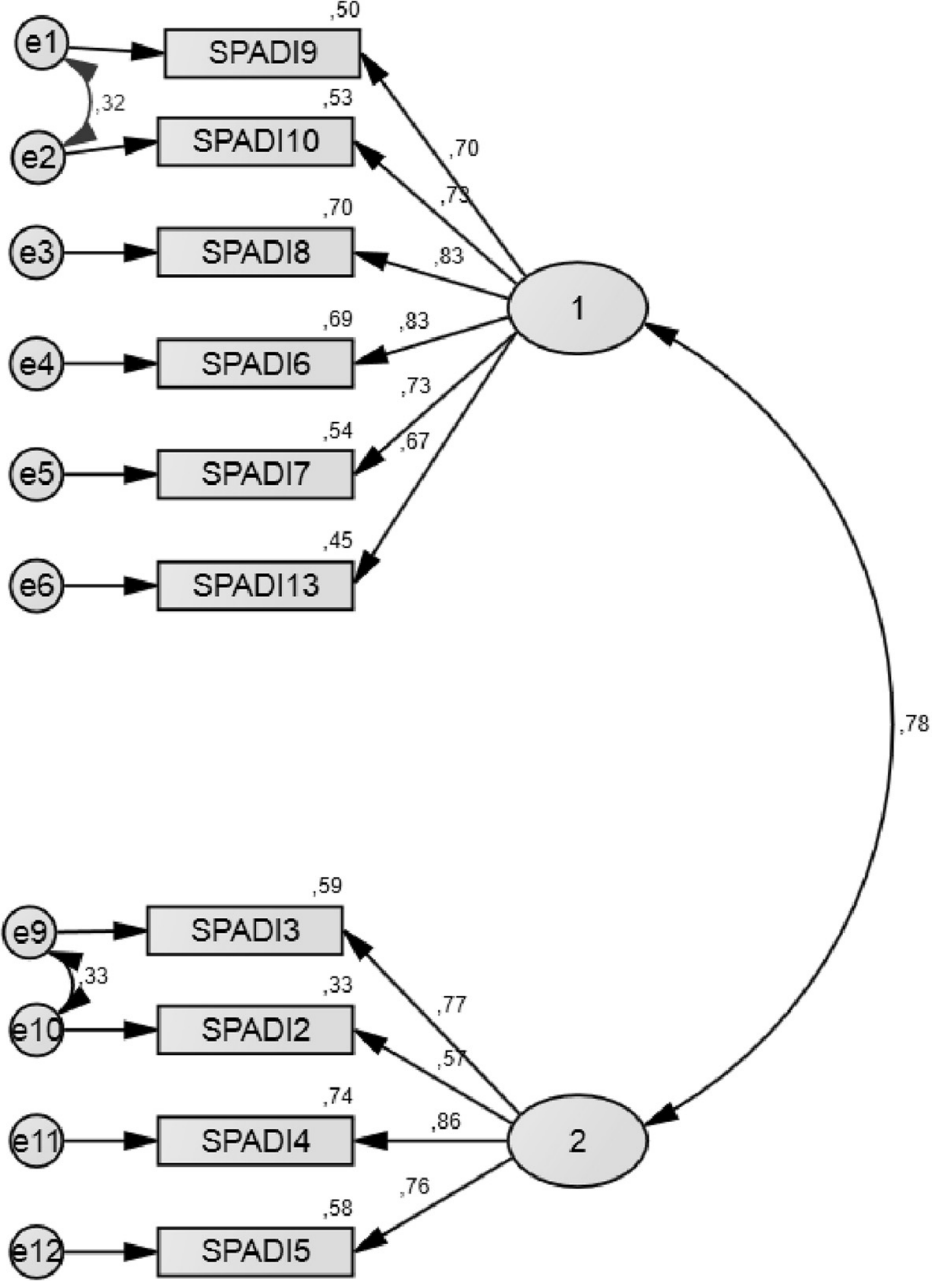
Confirmatory factor structure of SPADI

The original SPADI factor structure was tested by CFA, obtaining a poor fit: relative chi-square (*x*^2^/df) 3.16, CFI 0.89, NFI 0.92, and RMSEA 0.10 (90 % CI 0.08 to 0.12). An additional model was tested, after deleting items which have had a bad adjustment in the model (1, 11, and 12), obtaining the best fit: relative chi-square (*x*^2^/df) of 1.94, CFI 0.98, NFI 0.95, GFI 0,95, and RMSEA 0.06 (90 % CI 0.04 to 0.09) (Fig. 1). The analysis confirmed the bidimensional structure (pain and disability subscales).

Multinormality test was evaluated with Mardia’s coefficient, which obtained a value of 36.3. The global inter-item correlations of this new 10-item version were 0.55, with a Cronbach’s alpha of 0,90. Item-total statistics are shown in Table 2, being all of them above 0.5.

**Table 2:** Inter-item total statistics

	Scale mean if item deleted	Scale variance if item deleted	Corrected item-total correlation	Squared multiple correlation	Cronbach's alpha if item deleted
SPADI2	49,85	424,696	.515	.430	.908
SPADI3	49,53	415,248	.678	.603	.899
SPADI4	50,78	398,164	.724	.600	.896
SPADI5	50,39	404,958	.650	.520	.900
SPADI6	51,25	383,372	.754	.633	.894
SPADI7	49,19	408,582	.671	.527	.899
SPADI8	50,89	397,198	.755	.630	.894
SPADI9	53,35	403,221	.644	.562	.901
SPADI10	53,21	397,261	.687	.587	.898
SPADI13	51,18	393,603	.636	.426	.902

The convergent reliability between SPADI and DASH obtained a correlation Spearman’s Rho coefficient of 0.752 (*p* < 0.0001) and an intraclass correlation coefficient of 0.702 (*p* < 0.0001).

**Fig. 2:**
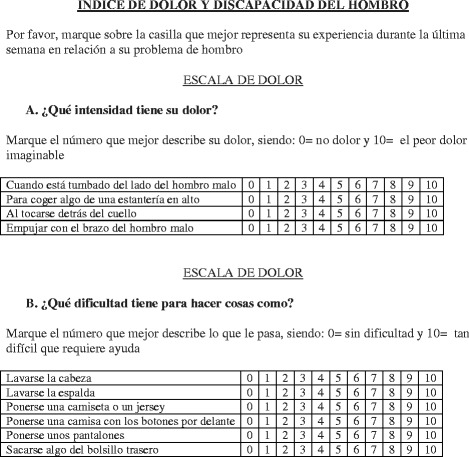
10-items Spanish language version of the SPADI

The final 10-items Spanish version of SPADI is shown in Fig. 2.

## Discussion

This study aimed to carry out the cross-cultural adaptation of SPADI for Spanish population and, secondly, to determine the psychometric properties of this version, in a population of patients suffering from shoulder pain. Regarding the first objective, the cross-cultural adaptation, our study encountered a few problems during the translation process, primarily in related to time period reference in the instructions. These problems were solved by the research team and the original author. The participants had no difficulty comprehending the questionnaire during the pilot study, and the translated version demonstrated good results in readability tests. We modified the original 13 item English language version of the SPADI by deleting those items with low correlations (items 1, 11 and 12). The confirmatory factor analysis for this new version attained the best fit. There was a strong correlation between the new Spanish Language SPADI and the Quick-Dash demonstrating good convergent validity. One of the strengths of this study was the scientific rigour of the methods used during the cross-cultural adaption phase. This included involving the original author to ensure the accuracy of the translation process. A previous study to adapt a new Spanish version of SPADI has been done recently [17]. These authors did not find any significant problems and/or difficulties in the cross-cultural adaptation process. The lack of difficulties in comprehension for the participants during the cross-cultural process is consistent with other studies [17, 28, 29]. Moreover, our new Spanish language version achieved good readability. The time required to complete the new Spanish Language SPADI are similar to the original version [11], although other cross-cultural versions have reported even less time to complete [29].

Regarding the psychometric properties, this is the first study of a Spanish language version of SPADI to utilize a factor structure analysis, with confirmatory methods. As a result of our structure factor analysis, items 1 (“How severe is your pain at your worst”), 11 (“Placing an object on a high shelf”) and 12 (“How much difficult do you have carrying a heavy object of 10 pounds”) were eliminated to achieve the best adjustment for the model. As a result, the new Spanish version for SPADI has different pain and disability subscales than the original English language version. Other studies have reported a unidimensional structure, without differences between pain and disability subscales [30, 31], while others were in consistent with the original version (two subscales) [32]. Based on our results, we recommend the use of this new 10-item Spanish SPADI-version.

The internal consistency of our version of the SPADI was good (Cronbach’s alpha of 0.90) was consistent with the findings of Torres-Lacomba et al [17], who also reported good values in internal consistency (0.96). It is important to note that the sample for Torres-Lacomba study was very different from the sample for our study in that it was drawn from women with shoulder pain after breast cancer surgery. Similar to other cross-cultural studies on SPADI [30–32], we found a strong correlation between the SPADI and the Quick-DASH indicating good convergent validity. Alsanawi et al [33] found a correlation between the SPADI and DASH of 0.84 (Spearman coefficient), while Ebrahimzadeh et al [28] reported a correlation of 0.61.

Our results imply that a new 10-items Spanish version for SPADI could be used by researchers and clinicians as a self-reported disability measure in patients suffering from shoulder pain in both routine clinical practice or in clinical research trials, This version keeps the properties of the original version, with two subscales (pain and disability). Spanish language is one of the most spoken languages around the world. Even though different Spanish speaker countries present some differences, semantic and grammatical rules are homogeneus, so that the version presented in this study could be of reference for other cross-cultural studies on SPADI, requiring only minor changes to adapt wording to accommodate the specific terms used in informal language. This new index could permit comparisons between other countries when studies on shoulder pain will be carried out.

However, there are some limitations in this study that should need to be recognized. Firstly, psychometric properties for Spanish-SPADI such as test-retest reliability, sensibility to change, as well as divergent validity have not been determined. The variety of different shoulder pain conditions in the participant sample could mean a risk of bias. Nevertheless, recent literature [34] recommends to avoid the use of subgroups in shoulder pain due to the lack of a gold standard for each of the diagnostic labels. The present study did no distinguish between participants with acute versus chronic shoulder pain and this might have influenced our findings so they must be taken with caution. Future investigations should be conducted to determine cutpoints in the score of this new version that could be used to classify patients with mild, moderate and/or severe shoulder pain.

## Conclusions

This study carried out a cross-cultural adaptation and validation of Spanish language version of the SPADI for Spanish population, and an examination of the psychometric properties of this new version. This study validated a new 10-items version of SPADI for Spaniards providing a patient reported outcome measure for use in this population in both clinical practice and research.

### Ethics approval and consent to participate

Ethics Committee of Costa del Sol in March 2014, Spain, approved this study (n° 011_marzo_PR). All participants in the study gave a written informed consent.

## Abbreviations

CFA: confirmatory factor analysis
CFI: the comparative fit index
CI: confidence intervals
DASH: disability of arm, shoulder and hand questionnaire
GFI: goodness of fit index.
NFI: normed fit index
PRO: patient reported outcome
RMSEA: root mean square error of approximation
SPADI: shoulder pain and disability index
